# Positional Information Is Reprogrammed in Blastema Cells of the Regenerating Limb of the Axolotl (*Ambystoma mexicanum*)

**DOI:** 10.1371/journal.pone.0077064

**Published:** 2013-09-27

**Authors:** Catherine D. McCusker, David M. Gardiner

**Affiliations:** Department of Developmental and Cellular Biology, University of California Irvine, Irvine, California, United States of America; University of Dayton, United States of America

## Abstract

The regenerating region of an amputated salamander limb, known as the blastema, has the amazing capacity to replace exactly the missing structures. By grafting cells from different stages and regions of blastemas induced to form on donor animals expressing Green Fluorescent Protein (GFP), to non-GFP host animals, we have determined that the cells from early stage blastemas, as well as cells at the tip of late stage blastemas are developmentally labile such that their positional identity is reprogrammed by interactions with more proximal cells with stable positional information. In contrast, cells from the adjacent, more proximal stump tissues as well as the basal region of late bud blastemas are positionally stable, and thus form ectopic limb structures when grafted. Finally, we have found that a nerve is required to maintain the blastema cells in a positionally labile state, thus indicating a role for reprogramming cues in the blastema microenvironment.

## Introduction

For over a century, regeneration biologists have puzzled over the ability of the regenerating region of an amputated salamander limb, known as the blastema, to replace exactly the lost distal structures. For example, amputation of a hand will result in the regeneration of a hand, while amputation through the upper arm will result in the generation of upper arm, forearm, and hand. This simple observation demonstrates that the cells of the stump have information about their position along the limb axis, and that they can access this information to determine what parts of the limb have been lost as a result of amputation [1,2]. By definition, regeneration is the replacement of the missing structures by the cells in the remaining stump. For this to occur the blastema cells derived from cells with proximal positional information need to be reprogramed to acquire new, more distal positional identities in order to replace the missing pattern.

The property of positional information is widely recognized in regeneration research as evidenced through the formation of supernumerary structures [1,2]. Cells with positional information are localized within the connective tissue [1-5], and use this information to control growth and pattern formation. When cells that are normally non-adjacent (i.e. come from different positions and thus have different positional information) are grafted next to each other, their subsequent interactions lead to proliferation and the formation of new pattern that normally lies between those cells. This process of pattern formation is referred to as “intercalation” [1,2,6,7], and not only leads to reestablishment of the proximal-distal (P-D) limb axis during regeneration [8], but also appears to generate the P-D limb axis during limb development in the mouse embryo [9]. In some instances, intercalation results in formation of the normal pattern, but in others can lead to formation of supernumerary limb structures.

Experimentally, the stimulation (or lack of stimulation) of supernumerary structures by intercalation allows for the identification of the presence and distribution of positional information encoded by cells from different locations within the limb [4,10,11]. In this study we used the formation of supernumerary limb structures in response to grafted cells from different stages of blastemas (early vs. late) and from different regions of blastemas (apical vs. basal) to determine whether blastema cells have positional information that is the same as (no supernumerary structures are formed) or is different from (supernumerary structures are formed) the host cells. We have focused on the question of whether blastema cells always have positional information that corresponds to their P-D level of origin, or if and when that information changes during blastema formation.

The likely answer to this question is that positional information is stable over long periods of time in the uninjured limb so that it is available when needed to regenerate new pattern, yet can be regulated dynamically (labile) so that cells with proximal information can give rise to cells with new, more distal information to replace the missing, more distal limb pattern. In 1901, T.H. Morgan hypothesized that blastema cells become developmentally labile (reprogrammable) in terms of their positional identity, and that they acquire new, more distal information as a result of interactions with more proximal limb stump cells that have an identity that coincides with the level of amputation. Consequently, the blastema cells immediately adjacent to the stump acquire new positional information that is more distal and thus replaces the missing pattern at the next most distal level. These newly re-patterned blastema cells then provide more distal information to reprogram the adjacent, more apical blastema cells that they are in contact with, and so on until replacement of the entire missing distal structure is completed [12].

This model of how more proximal cells with stable positional information progressively reprogram positionally labile blastema cells is consistent with a number of observations from blastema transplantation studies. Experiments independently performed by P. Weiss, B.D. Milojevic, G. Schwidefsky, and S.V. Bryant on different stage blastemas led to the interpretation that early stage, or ‘undifferentiated’ blastema cells acquired new limb pattern by interacting with cells at the new host site; whereas, cells from later stage blastemas became progressively refractory to being reprogrammed, and thus regenerated limb patterns corresponded to the position of origin of the graft [13-16].

This early interpretation of the results from experiments in which later stage blastemas were grafted led eventually to the model of the late-stage blastema as a self-organizing system such that when grafted to an ectopic host site, an entire new limb would form [17]. Thus by the late blastema stage, the P-D identity of the blastema cells apparently had become stability reprogrammed. In contrast, when early stage blastemas were grafted, ectopic limb structures typically were not formed. Contrary to the earlier interpretation that these cells do not form supernumerary structures because they are undifferentiated (positionally labile), an alternative interpretation was proposed such that grafted early blastema cells are lost through “resorption” and replaced by host cells with positional information corresponding to the proximal-distal level of the amputated stump tissues [17-23]. Until recently it has not been possible to trace precisely the fate of the grafted blastema cells because of the lack of unambiguous labeling techniques [21,23], and thus it has not been possible to test the “resorption” hypothesis.

While conducting experiments that involved grafting blastema cells to ectopic wounds on the side of the arm of axolotls [11,24], we observed that grafted early blastemas failed to form ectopic limbs as anticipated based on the “self-organizing system” model [25]. We thus revisited the issue of how the limb pattern is reestablished in the blastema by grafting blastema cells from transgenic animals expressing Green Fluorescent Protein (GFP) to non-GFP hosts to follow the fate of the grafted cells and their progeny throughout the process of regeneration (Figure S1). We have discovered that the cells from early stage blastemas typically survive and their progeny contribute to the regenerated limb structures even though no supernumerary limb structures are formed. This finding is consistent with the hypothesis that early blastema cells are developmentally labile such that their positional identity is reprogrammed to form the limb pattern that is appropriate to the host site. We have discovered that at later blastema stages, cells in the apical region also are positionally labile but that cells in the basal region have reacquired a new positional identity and become positionally stable. Finally, we have found that a nerve is required to maintain blastema cells in a positionally labile state, thus indicating a role for reprogramming cues in the blastema microenvironment.

## Materials and Methods

### Ethics Statement

This study was carried out in accordance with the recommendations in the Guide for the Care and Use of Laboratory Animals of the National Institutes of Health. The experimental work was approved by the Institutional Animal Care and Use Committee of the University of California Irvine (Protocol # 2007–2705).

### Animals

All of the experiments in this study were performed on small to medium-sized Mexican axolotls ( *Ambystoma* *mexicanum* ) measuring approximately 10-15 cm from snout to tail tip (5-7 cm snout to vent) either spawned at UC, Irvine or obtained from the Ambystoma Genetic Stock Center, University of Kentucky. Animals were anesthetized using a 0.1% solution of MS222 (Ethyl 3-aminobenzoate methanesulfonate salt, Sigma), pH 7.0. To initiate regeneration, animals were either amputated just proximal to the carpals (distal amputation), or at the proximal end of the humerus (proximal amputation).

### Surgical procedures

**Figure 1:**
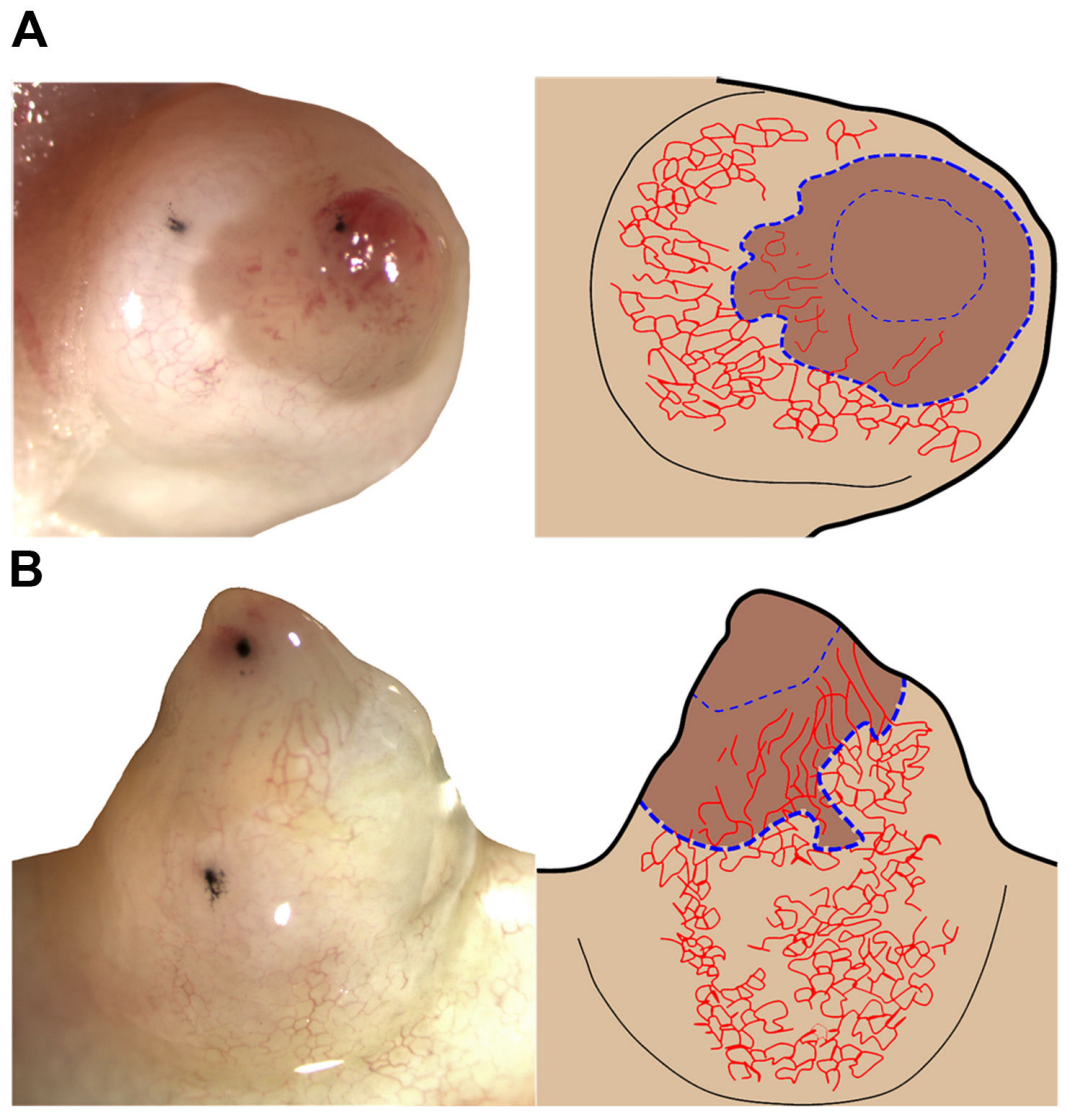
Description of early blastema and apical late blastema tissue grafts. Images on the left are of an early bud (EB) blastema (**A**) and a late bud (LB) blastema (**B**). Cartoons to the right highlight landmarks in the EB blastema and LB blastema that were used to determine where the graft (donor) tissue was taken from. (**A**) Distal view of an EB blastema, where carbon (dark spots) was used to mark anterior stump and anterior blastema. The intact basement membrane made the stump appear lighter in color than the blastema tissue, which was covered with a wound epithelium and did not have a basement membrane. Additionally, the blood vessels in the stump were highly branched (honeycomb), while the blastema tissue had fewer visible blood vessels that aligned along the proximal/distal axis. The thick blue dotted line indicates the boundary between the stump tissue and the early blastema tissue. The thin blue dotted line marks the proximal boundary of the graft. (**B**) Anterior view of a late blastema with carbon marks identifying the anterior stump, and anterior apical tissues. The basal region of the LB blastema typically had blood vessels that were aligned along the P/D axis, while the apical region had very few visible blood vessels. The apical graft tissue (thin blue dotted line) was taken above these blood vessels to decrease the chances of including basal tissue.

Early bud (EB) blastema donor tissue was obtained from either proximal or distal amputation sites on a transgenic animal expressing GFP. To avoid including stump tissue, we were carful to take only the region of the blastema where blood vessels were not visible (see Figure 1A for an example of the typical EB blastema graft). The EB blastema tissue was then grafted to distal or proximal level amputations on non-GFP hosts that were at the same stage of regeneration (Figure S1). The blastema on the host limb was removed before grafting GFP donor blastemas. To allow the graft to adhere to the host site, animals were kept on ice for 2 hours, misting with 40% Holtfreter’s every 30 minutes.

Grafts of the apical and basal region of late bud (LB) blastemas were performed as described for EB blastemas. Apical blastema grafts were obtained from the tip of the LB blastema where no blood vessels were visible (Figure 1B). The observation that the tip of the blastema exhibits minimal vasculature has been characterized previously [26]. Basal blastema grafts were obtained from the region of the LB blastema closest to the stump tissue. Basal graft tissue was obtained conservatively so as to avoid including cells from the stump. Basal grafts were trimmed so that the size of the graft was roughly the same as apical grafts. In most cases, GFP fluorescence from the grafted tissue was visible under a dissecting scope until the completion of the experiment (up to 10 weeks).

The wound epithelium was not removed from EB or LB tissue grafts. Thus, the EB and apical-LB grafts included the Apical Epithelial Cap (AEC). The basal-LB grafts included the basal wound epithelium, which is not likely to include much of the AEC. However, it has been shown that the wound epidermis only takes a few hours to cover an exposed surface on the axolotl limb [27,28]. Additionally, Thornton showed that the AEC regrows after it has been removed from the blastema [29]. Thus, it its likely that the exposed surface of the basal-LB graft (where the apical region had been removed), was rapidly covered with a new AEC shortly after grafting.

**Figure 2:**
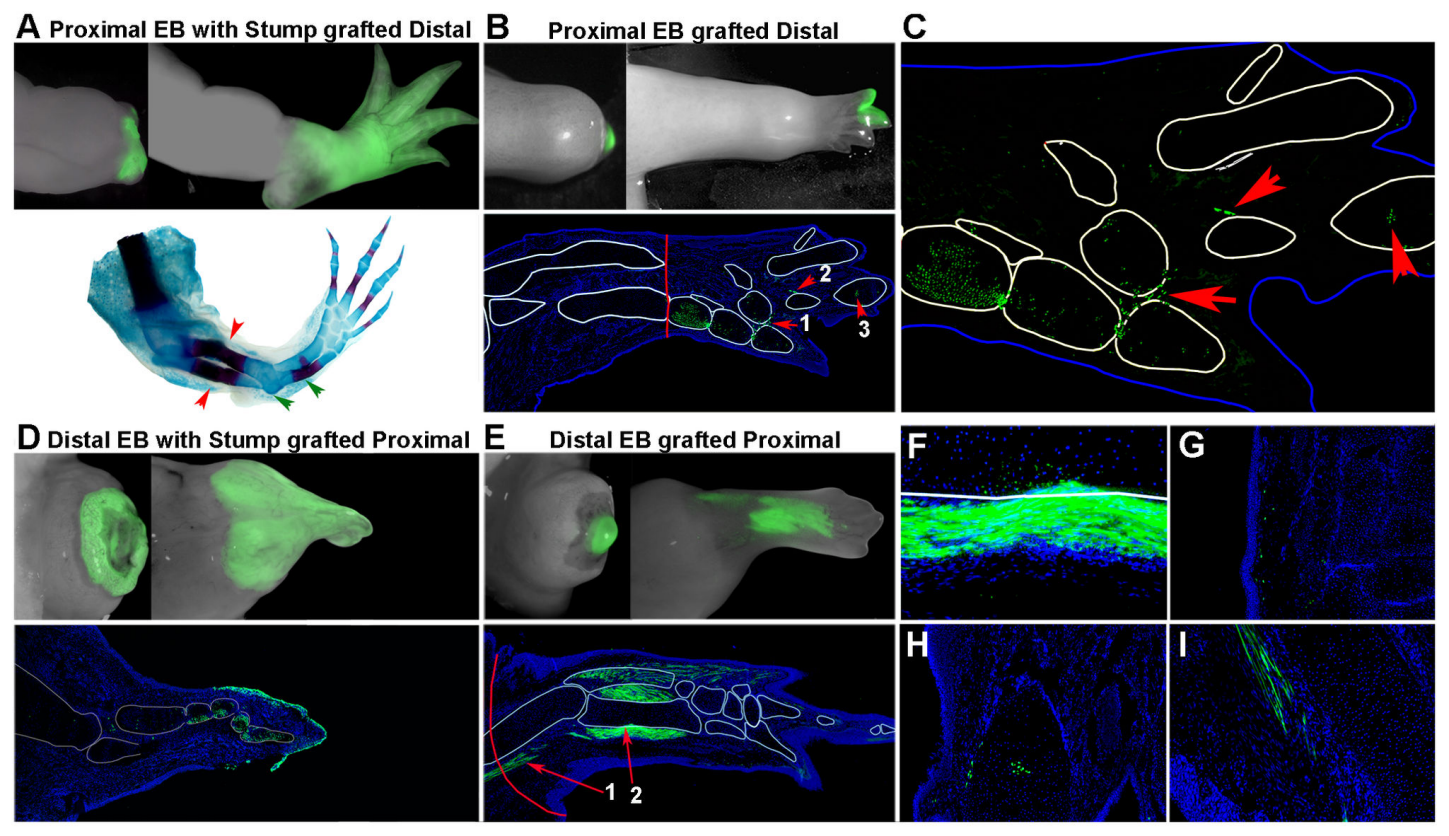
Grafted early bud blastema cells survived but did not form supernumerary limb structures (**A**-**C**) Proximal EB blastemas grafted to the stump of a limb amputated at a distal level. Graft cells contributed to connective tissue (**A**) Live images of proximal EB blastema *with* stump grafted to the stump of a limb amputated at a distal level, 1 and 5 weeks post surgery, and whole mount skeletal preparations from 7 weeks post surgery. Red arrows indicate the distal amputation plane on the host limb. Green arrows point to extra elbow joint and radius/ulna. Duplicated proximal structures were observed in 5/6 grafted limbs. (**B**-**C**) Proximal EB blastemas *without* stump grafted to the stump of a limb amputated at a distal level. None of the grafted blastemas (6/6) formed duplicated proximal-distal limb patterns. (**B**) Live images at 1 and 5 weeks post surgery, and tissue section 7 weeks post surgery where the regenerated skeletal tissues are outlined in white. Nuclei are stained with DAPI (blue), and grafted cells are GFP positive (green). Red line demarks the host amputation plane (higher magnification in (**C**)). The grafted cells contributed to a variety of different tissues including connective tissue (arrow 1), muscle (arrow 2), and cartilage (arrow 3). (**D**-**I**) Distal EB blastemas grafted to the stump of a limb amputated at a proximal level. (**D**) Live images of distal EB blastema *with* 1 mm of stump grafted to the stump of a limb amputated at a proximal level. The grafted cells remain viable and contributed to regenerated tissues as evident in sections of regenerated limbs 7 weeks post surgery in all grafts with (5/5) or without (**E**) (8/8) stump tissue included. Grafted cells contributed to nerves (arrow 1), bone (arrow 2, magnified in **F**), fibroblast-like cells in the dermis (**G**) connective tissue surrounding cartilage and cartilage (**H**), and muscle (**I**).

**Figure 3:**
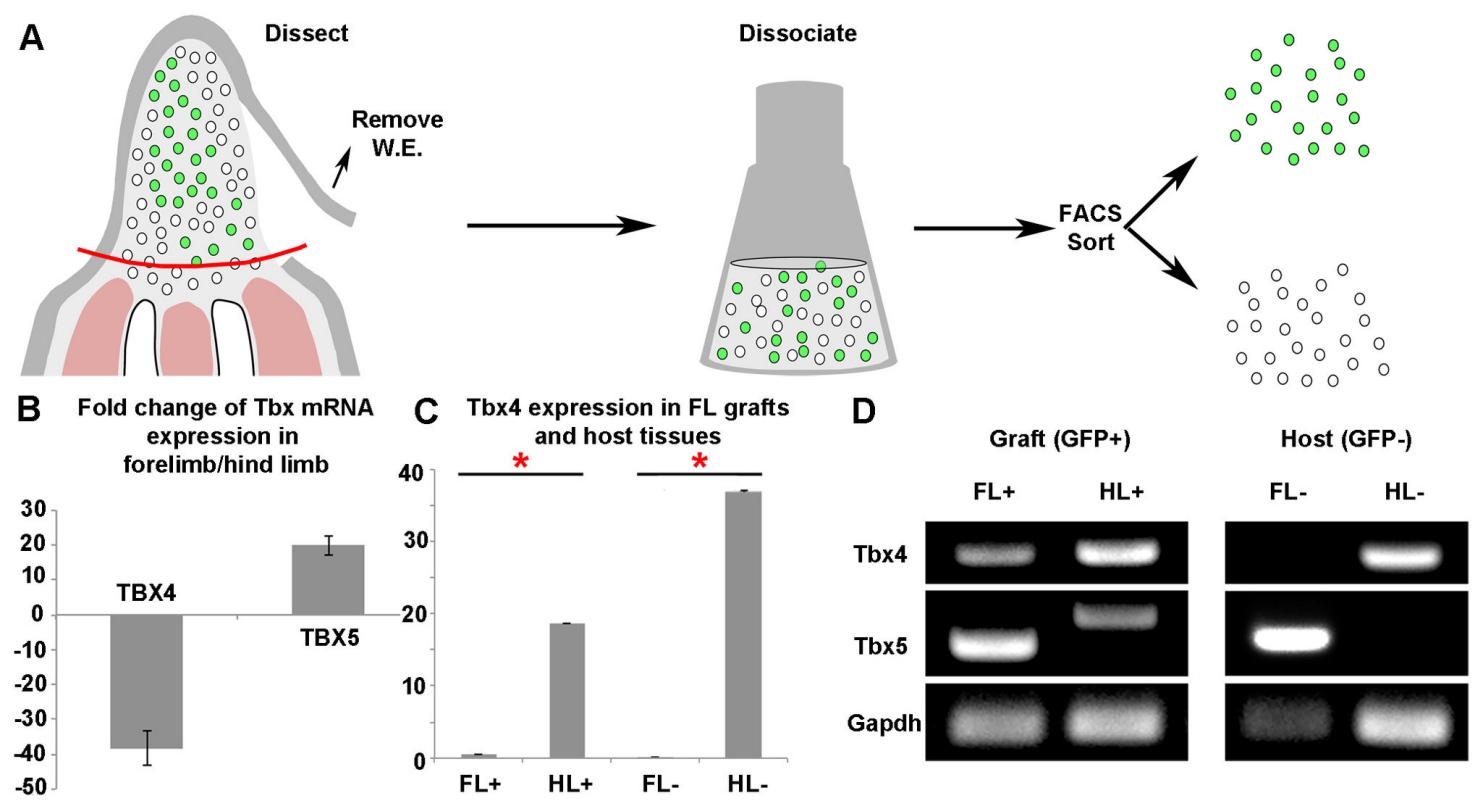
Forelimb (FL) EB blastema cells were reprogrammed and expressed hind limb markers when grafted to the stump of an amputated hind limb (HL). (**A**) Illustration describing how GFP+ (graft) cells were isolated from GFP- (host) cells in a mosaic blastema created by grafting a GFP+ blastema to a GFP- host. (**B**) Validation by q-rtPCR of *Tbx4* and *Tbx5* as markers for HL and FL respectively. Histogram is fold change of expression in FL blastemas relative to HL blastemas. Error bars are SE (N = 3 technical replicates). (**C**) Representative histogram of relative *Tbx4* expression in GFP+ FL grafts to FL or HL (FL+, HL+, respectively) and GFP- FL or HL host tissue (FL-, HL-). Error bars are SEM (N = 3 technical replicates). P-values were determined by T-test with 2 tails and unequal variance (N = 3 biological replicates for which cells were pooled from 8 blastemas for each sample). (**D**) RT-PCR (35 cycles) for *Tbx4, Tbx5*, and *GAPDH* performed on graft and host cells. The sequence of the high molecular weight *Tbx5* band can be found on GenBank (Accession # KC920480). Illustrations describing the surgical manipulations performed in this study are in Figure S1C,D.

**Figure 4:**
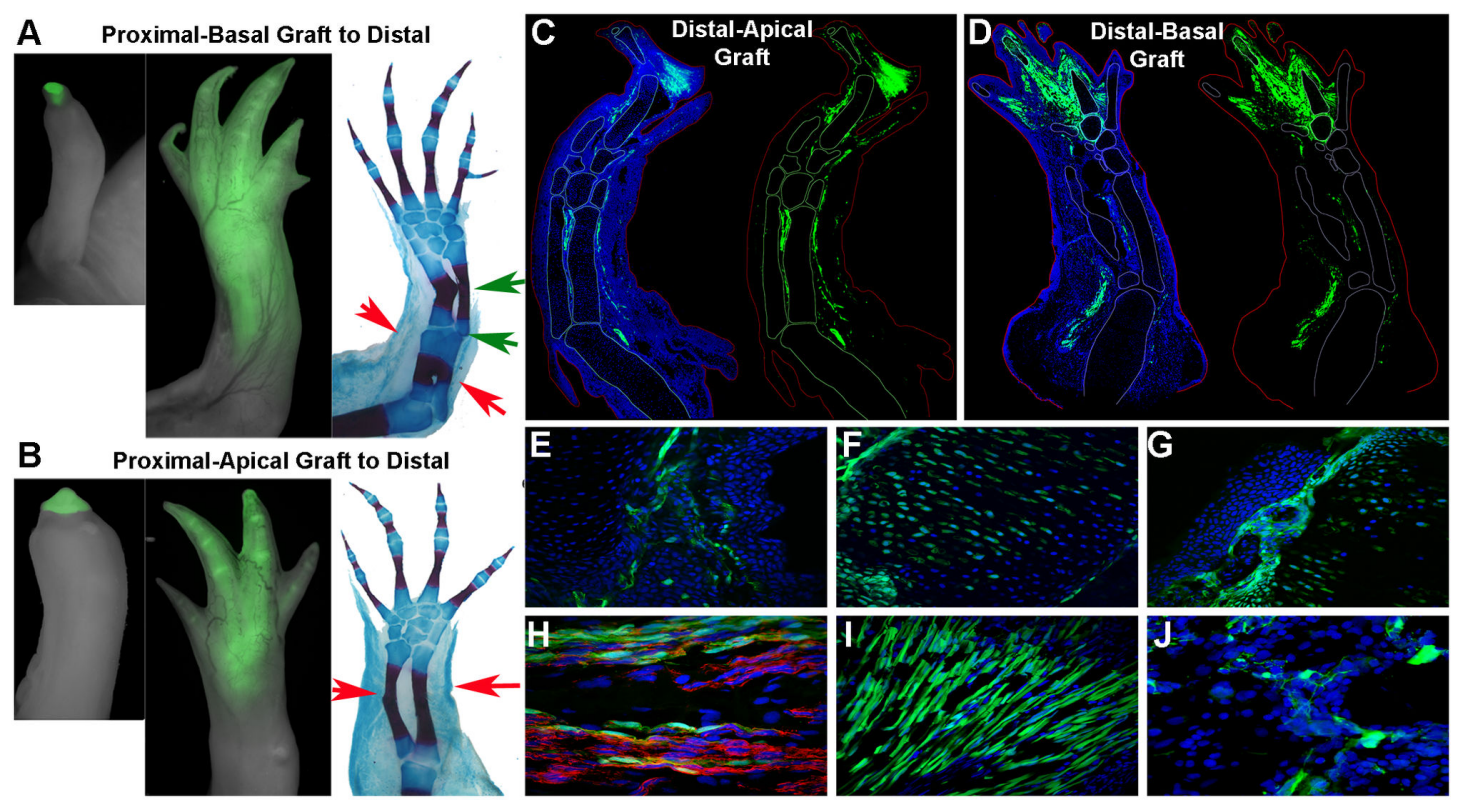
Apical and basal regions of LB blastema grafts had different levels of positional stability/lability. (**A**) The basal region from a proximal LB blastema grafted to the stump of a limb amputated at a distal level (imaged 1 and 7 weeks post-grafting) gave rise to a limb with a duplicated proximal-distal limb pattern as evident in whole mount skeletal preparations. Red arrows indicate the distal amputation plane on the host limb. Green arrows point to extra elbow joint and radius/ulna. Duplicated proximal structures were observed in 5/6 grafted limbs. (**B**) In contrast, when the apical region from a proximal LB blastema was grafted as in (**A**), almost no proximal pattern duplications were observed (N=5/6). (**C**) Longitudinal section of a regenerated limb derived from apical LB or basal-LB (**D**) blastema grafts from a distal amputation (green) to a proximal host site. GFP+ graft cells live and differentiate into a variety of tissues in the regenerate (6/6 apical grafts, 5/5 basal grafts). (**E**-**J**) Grafted cells from the apical and basal region of a LB blastema contributed to a variety of tissues including (**E**) blood vessels, (**F**) cartilage, (**G**) connective tissue surrounding skeletal elements, (**H**) nerve-associated cells (red stain is for acetylated β-tubulin in neural axons), (**I**) muscle, and (**J**) cells in the dermis.

To ensure that the graft had not fallen off or been rejected by an immune response, we grafted tissues from transgenic animals expressing Green Fluorescent Protein (GFP). While the majority of grafts integrated into the host site (representative images in Figures 2-4), a few grafts did not heal to the host site or disappeared from the host site shortly after grafting. Thus, grafts that were not visible for at least three weeks following surgery were not included in subsequent analyses.

The induction of ectopic blastemas was performed as described previously [11]. For experiments in which blastemas were grafted to a host wound site on the side of the arm, the donor blastema was grafted immediately after the brachial nerve was deviated.

Blastemas from limbs lacking innervation were obtained by surgically severing the brachial nerves innervating the limb three days prior to the surgery to remove the donor blastemas as detailed above [30]. The contralateral limbs with the nerves intact were used as donor limbs for innervated blastemas.

### Cell dissociation, sorting, and RNA isolation

Forelimb (distal) blastemas from transgenic animals expressing GFP were grafted to the forelimb (distal) stump or hind limb (distal) stump of a white animal (Figure S1C-D). When the chimeric blastemas reached LB stage, they were harvested for cell sorting (n=8 blastemas per graft scenario). The wound epithelium was removed manually, and the chimeric blastema mesenchyme was dissected from the stump. To remove the contaminating red blood cells, the blastemas were rinsed with di H2O and washed in 40% Holtfreter’s solution, and remaining RBCs were removed manually with forceps. To form a single cell suspension, the blastemas were teased apart with forceps and incubated in a 3000 U/ml collagenase (Fisher) solution in 60% L15 media (Sigma-Aldrich) for 3 hours at room temperature with agitation. The cells were pelleted at 1000 G for 5 min, resuspended in 1xPBS, passed through a 70µM filter, and kept on ice with agitation until loading into the Dako Cytomation MoFlo Cell Sorter. Forward and side scatter were used to gate all cells from cellular debris. Pure white (GFP-) and pure GFP (GFP+) populations were run to establish gates for GFP+ and GFP- cells. The difference in the mean fluorescence intensity of GFP- and GFP+ cells was 1000 fold, with peaks at 10° and 10^3^ mean fluorescence intensity respectively, in the green fluorescent channel (Figure S2). To ensure that minimal contaminating (GFP-) cells were included in the grafted (GFP+) cell population, the sorting gates were set conservatively around the GFP+ population, excluding cells below 10^2^ mean fluorescent intensity (Figure S2C).

To assess the relative amount of GFP+/GFP- doublets that were present in the GFP+ population, we analyzed the area *versus* the width and the area *versus* height of each particle in the fluorescent channel, as performed previously [31], using a FACSCalibur flow cytometer (BD Biosciences). The relative amount of GFP+/GFP- doublets from three independent samples was determined to be 01.36% +/- 0.311% (A vs. W) (Figure S3).

GFP+ and GFP- cell populations from grafted blastemas were sorted directly into TriPure (Roche) and extracted with ChCl3, after which 70% EtOH was added to the aqueous layer before using a Nucleospin RNA XS kit (Machery-Nagel, Bethlaham, PA) to purify the RNA. The number of GFP+ cells sorted for each sample was 2363 +/- 581, and 3071 +/- 608 cells for FL grafted to FL, and FL grafted to HL samples, respectively (n =3 independent experiments).

### RT-PCR and QPCR

The reverse transcription reaction and DNAse treatment were performed using the Transcriptor First Strand cDNA Synthesis Kit (Roche, Mannheim, Germany) following the manufacture’s protocol. Control cDNA was obtained from limb mesenchyme tissue including dermis, muscle, cartilage, and nerve. A 1:10 serial dilution of the control cDNA was used to calculate the efficiency of each primer set as described in the Pffafl method [32]. Both GAPDH and EF1α were used as normalizing genes. Q-rtPCR reactions were performed using Cybergreen master mix (Roche) in a Light Cycler 480 II (Roche). Relative quantification of *Tbx4* transcript expression was calculated using the Pfaffl method, where experimental Cp values were compared to control Cp values for each primer set. The data for each sample were represented as a ratio of the relative expression of *Tbx4* to the relative expression of the reference gene (*EF1α*). QPCR and RT-PCR primer sequences were follows:

GAPDH Forward: 5’-TCTTCCAGGAGCGTGACCCC
GAPDH Reverse: 5’-GCACCTCTGCCATCTCTCCACAG
EF1α Forward: 5’-CGGGCACAGGGATTTCATC
EF1α Reverse: 5’-TGCCGGCTTCAAACTCTCC
TBX4 Forward: 5’-AGCCAATGAGTTCCTATACGCCCA
TBX4 Reverse: 5’-AAAGGACAGTCATCCATCCGTCCA
TBX5 Forward: 5’-CTGGAAGGCGCATGTTTCCAAGTT
TBX5 Reverse: 5’-TGGCGAATCCGGATGGACGTATAA

*Ef1α* primer sequences were used as in [33], *GAPDH* primer sequences were as in [34]. *Tbx4* and *Tbx5* primers were based on the sequences from The Ambystoma EST Database (contig83419 and contig99801, respectively).

### Tissue preparation and whole mount bone and cartilage staining

Tissues were fixed in 3.7% PFA and prepared for cryosectioning. The anti-acetylated β-tubulin antibody (Sigma) was used as described in [35]. The sections were stabilized with Vectashield mounting medium with DAPI (Vector Laboratories, Burlingham, CA). Images were obtained using a 20x objective on a Zeiss LSM780 (2-photon) confocal microscope. Whole-mount Alcian blue and Alizarin red staining of limbs was performed as described in [36].

## Results

### Grafted early bud blastema cells survive but do not form supernumerary limb structures

To test whether the cells of the early blastema have stable positional information or if they are positionally labile, we grafted proximal EB blastemas to distal amputation stumps to observe whether regenerates with duplicated proximal-distal (P-D) structures formed (Figure 2A-C, Figure S1A). We initially confirmed [23] that regenerates with duplicated proximal/distal structures resulted when proximal EB blastemas *with* stump tissue were grafted to a distal amputation (5 of 6 grafted limbs exhibited P-D pattern duplication) (Figure 2A). These limbs had a duplicated elbow and proximal zeugopod (Figure 2A, green arrows) in addition to duplicated structures where the host zeugopod and the grafted stylopod patterns were fused (Figure 2A, region distal to the red arrows).

In contrast, when proximal EB blastemas *without* stump tissues included were grafted to a distal amputation host a regenerate with the normal P-D pattern formed (6 of 6 grafted limbs were normal) (Figure 2B-C). This finding was consistent with the interpretation that early stage blastema cells were labile and acquired new P-D positional information by interacting with cells at the new (distal) host site [13-16]. The presence of stump tissues in the EB blastema grafts presumably provided P-D information corresponding to the level of the donor tissues (proximal) from which the EB blastema cells formed more distal pattern [1,2,37] resulting in a limb with a duplicated P-D pattern. Thus the grafted cells would have regenerated the pattern distal to the grafted proximal stump tissues, and the host cells would have intercalated the missing pattern between the distal host and grafted proximal stump tissues (Figure 2A).

In contrast to the situation in which a proximal blastema was grafted to a distal stump, the reciprocal grafting combination (distal blastema grafted to a proximal host) would not be expected to lead to the formation of supernumerary structures (Figure S1B). As anticipated, all regenerated limbs had a normal pattern when distal EB blastemas were grafted whether or not stump tissues were included in the graft (5 of 5 grafts *with* stump included; 8 of 8 grafts *without* stump included) (Figure 2D-E).

GFP-positive cells from both proximal EB blastemas and distal EB blastemas survived after being grafted, and their progeny integrated into the host site and contributed to structures of the regenerate (Figure 2A-I). Thus neither proximal nor distal EB blastema cells were “resorbed” or lost when grafted. By morphological criteria, the progeny of the grafted cells contributed to a variety of tissues including cartilage, fibroblast-like cells in the dermis, loose connective tissue, cells within nerve bundles, and muscle (Figure 2E-I).

When distal blastemas were grafted to a proximal host stump, the regenerated tissues located between the proximal host amputation plane (red line in Figure 2E) and the distal donor amputation plane (see red line in Figure 2B) contained cells of both host and donor origins (Figure 2E,F). The contribution of distal blastema cells to more proximal structures was particularly evident when no stump tissues were included in the blastema grafts (Figure 2E); however, some distal cell contribution to more proximal structures was also observed when stump was included in the graft (compare Figure 2E and 2D). Although it had been reported that only proximal stump cells contribute to the intermediate region of the new pattern [21,38], recent studies have shown that at least some tissues including muscle, Schwann cells and epidermis from more distal regions of the limb can contribute to regeneration of more proximal regions of the limb [3,5,39]. Because at present there are no molecular markers for the connective tissue cells that have the property of positional information [3,5,40], it is not yet possible to identify and isolate those cells in order to study their behavior and contribution specifically.

### EB blastema cells express positional marker genes that are consistent with the host site to which they are grafted

To determine if EB blastema cells can express new positional-identity genes when grafted to a new limb position, we analyzed the expression of four genes that are expressed by blastema cells from different positions in regenerating limbs. To do this, we used FACS to recovered GPF-positive cells that had been grafted to a new host site and allowed to participate in regeneration. Distal forelimb EB blastemas from GFP donor animals were grafted to the proximal or distal stumps of either the forelimb (FL) or hind limb (HL) of a non-GFP host animal (Figure S1C, D). The chimeric blastemas were collected at the LB stage, dissociated, and the GFP-positive (grafted) cells were sorted from GFP-negative (host) cells by FACS (Figure 3A). We then used qRT-PCR to quantify differences in the level of gene expression of proximal/distal (*Hoxa-9* and *Hoxa-13*), and forelimb/hind limb (*Tbx5*, and *Tbx4*) marker genes [8,41,42].

Although *Hoxa-9* and *Hoxa-13* were co-expressed in both proximal and distal blastemas [8], there was a significant difference in expression levels such that *Hoxa-9* was expressed approximately 5x higher in proximal blastemas, and *Hoxa-13* was expressed approximately 1.5x higher in distal blastemas (data not shown). Given the limited yield of recovery of grafted cells by FACS, we determined that it was not possible to generate sufficient sample sizes to determine whether or not there was a statistically significant change in the relative levels of gene expression of these markers when blastema cells were grafted from distal to proximal. In contrast, *Tbx5* expression was 20x greater in forelimb (FL) blastemas, and *Tbx4* expression was 38x greater in hind limb (HL) blastemas (Figure 3B). Given this difference in *Tbx5/Tbx4* expression between forelimbs and hind limbs, we were able to utilize these markers to test whether grafted FL blastema cells were induced to express significantly higher levels of the HL marker (*Tbx4*) when grafted to a HL stump.

EB blastema cells with FL positional identity (low *Tbx4* expression and high *Tbx5* expression) changed their pattern of gene expression and expressed the marker for HL positional identity (high *Tbx4* expression) when grafted to a HL stump (Figure 3C,D). We note that a very low level of *Tbx4* was observed in the control samples (GFP+ graft cells from the FL grafted to FL stumps) (Figure 3C,D) which is consistent with the observation that ungrafted FL blastema cells also express *Tbx4* at a low level as compared to HL blastema cells in [43]. In comparison to these control grafts (FL cells grafted to a FL host), the level of *Tbx4* expression was significantly increased (27x +/- 13x for three experiments), and expression of the normal *Tbx5* transcript was not detected in FL blastema cells that had been grafted to a HL host (Figure 3C,D). We observed that the relative amount of GFP+/GFP- (graft/host) doublets was less than 1% of the GFP+ cell population (Figure S3). Thus, it is unlikely that the differences observed in *Tbx4* and *Tbx5* expression in the graft (GFP+) cell populations were a result of contaminating host (GFP-) cells.

We note that previous grafting studies between FL and HL blastemas have led to the conclusion that FL cells cannot be reprogramed into HL, and *vice versa*. As with the classic experiments in which blastemas were grafted between proximal and distal levels (the basis of the present study), many of these experiments were performed with either non-regenerating tissues [44] or with advanced stage blastemas [45,46]. For those studies in which early stage blastemas were grafted between FL and HL, the data for many of the grafts were excluded based on the assumption that the grafted cells had died or were “resorbed” [17,47]. The grafting and recovery of GFP+ cells by FACS in the present study precluded this possible interpretation.

Although the *Tbx5* amplimer that is characteristic of FL blastema cell identity was not detected in the FL cells grafted to a HL host, a novel *Tbx5* transcript was observed (Figure 3D). This amplimer contained an insertion of 184 base pairs with three in-frame stop codons between the exon sequences within the T-box domain, which would generate a truncated form of the *Tbx5* protein (GenBank accession # KC920480). Although we do not know how expression of this truncated variant of *Tbx5* might affect the grafted cells, we note that mutations of this region of *Tbx5* in humans are associated with Holt-Oram syndrome, which is characterized by forelimb and cardiac malformations [48].

### Grafted cells from the apical and basal regions of a late bud blastema differ in terms of their ability to induce formation of supernumerary limb structures

Given that the blastema continues to reform new pattern along the P-D axis throughout the period of regeneration, it is likely that a population of cells that can form new distal pattern is maintained through the later stages. As the regenerating blastema develops, it become spatially heterogeneous such that cells in the more basal region are beginning to differentiate at the same time that cells in the more apical region are still undifferentiated and appear similar to those of the early stage blastema [49-51]. We therefore hypothesized that the cells localized in the apical region of a late bud (LB) blastema from a proximal amputation would be comparable to EB blastema cells in terms of whether or not they formed supernumerary limb structures when grafted to a distal amputation stump (Figure S1A).

As observed with grafted proximal EB blastemas *with* stump tissue (Figure 2A), a limb with duplicated proximal-distal structures was regenerated when the basal region of a proximal LB blastema was grafted to a distal amputation (5 of 6 grafted limbs exhibited P-D pattern duplication) (Figure 4A). These limbs had a duplicated elbow and proximal zeugopod (Figure 4A, green arrows) in addition to duplicated structures where the host zeugopod and the grafted stylopod patterns were fused (Figure 4A, region distal to the red arrows). In contrast, in almost all cases a normal limb pattern was regenerated when the apical region of a proximal LB blastema was grafted distally (5 of 6 grafted limbs exhibited a normal P-D pattern) (Figure 4B). Thus the outcome of grafting apical LB blastema cells is the same as grafting EB blastema cells *without* stump tissue included; whereas, the outcome of grafting basal LB blastema cells is the same as grafting EB blastema cells *with* stump. This finding was consistent with the interpretation that apical LB blastema cells (as well as EB blastema cells) were undifferentiated and acquired new P-D positional information by interacting with cells at the new host site [13-16]. On the other hand, basal LB blastema cells have acquired stable P-D positional information and maintain their identity when grafted, which is consistent with the model of the late-stage blastema is a self-organizing system such that when grafted to an ectopic host site, an entire new limb would form [17].

As with EB blastema grafts, the reciprocal grafting combination (distal LB blastema grafted to a proximal host) resulted in regenerated limbs with a normal pattern when either the apical region (6 of 6) or the basal region (5 of 5) of distal LB blastemas were grafted proximally (Figure 4C,D, Figure S1B). GFP-positive cells from both apical and basal regions of both proximal and distal LB blastemas survived after being grafted, and their progeny integrated into the host site and contributed to structures of the regenerate (Figure 4A-J). Thus neither apical nor basal LB blastema cells were “resorbed” or lost when grafted. By morphological criteria, the progeny of the grafted cells contributed to a variety of tissues including cartilage, fibroblast-like cells in the dermis, loose connective tissue, cells within nerve bundles, and muscle (Figure 4E-J).

### The presence of a functional nerve is required to maintain EB and apical LB blastema cells in an undifferentiated state

The nerve is required for successful limb regeneration because it interacts with the wound epithelium to induce formation and maintain function of the apical epithelial cap (AEC) [24,52,53]. Signaling from the AEC recruits cells from the limb stump to accumulate below the wound epithelium, establishing the early blastema [11,35]. Signaling from a nerve is required until the late stages of regeneration, and if the blastema is denervated prior to this stage a hypomorphic, or distally truncated, regenerate will form [54,55]. Thus the nerve is thought to function in maintaining blastema cells in an undifferentiated and proliferative state [11].

**Figure 5:**
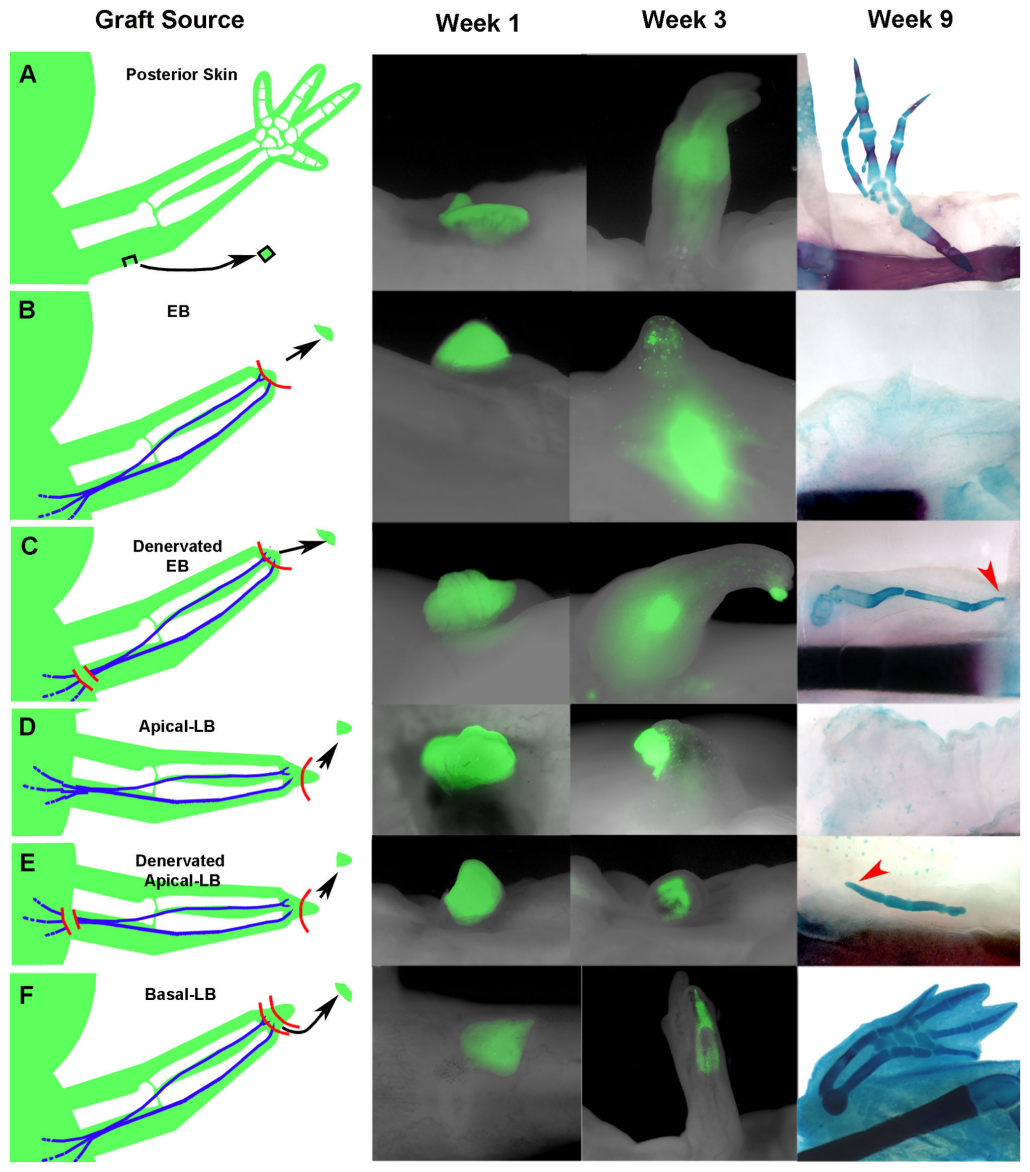
Innervation was required to maintain positional lability of EB blastema cells and apical-LB blastema cells. (**A**) An ectopic limb was formed when posterior skin was grafted to a nerve-deviated wound on the anterior side of the arm (7/9) [11]. Consistent with our previous results, 7 of 8 grafts of basal LB blastemas formed well-patterned distal limb structures (**F**); whereas, 25 of 30 grafts of either EB blastemas (**B**) and 14 of 17 apical LB blastemas (**D**) did not form ectopic cartilage structures. In contrast, 9 of 14 grafts of EB blastemas from denervated donor limbs (**C**) and 6 of 9 apical LB blastema grafts from denervated donor limbs (**E**) formed ectopic skeletal elements. Experimental limbs were imaged 1 week and 3 weeks post-grafting, and samples were stained for the skeletal patterns in whole-mount preparations (right) 9 weeks post-surgery. Red arrows in (**C**) and (**E**) indicate the distal tip of the ectopic structures. Illustrations describing the surgical manipulations performed in this study are in Figure S1E. A more detailed quantification of the regenerated phenotypes in the present study is presented in Table S1.

To test whether limb cells have stable positional information (differentiated) or labile positional information (undifferentiated) we grafted mature skin and blastema cells (EB, apical LB and basal LB) to wounds with a surgically deviated nerve on the anterior side of the arm (Figure 5 and Figure S1E) [11]. These wounds form an ectopic blastema but in the absence of grafted cells with positional information no ectopic limb structures form [11,24]. We confirmed that posterior skin (differentiated cells) grafted to the innervated wound site on the anterior limb led to formation of an ectopic limb (Figure 5A) [11]. Similarly, grafted basal LB blastema cells formed ectopic limbs (Figures 5F, 7 of 8), a result that is consistent with the formation of P-D duplicated limbs when these cells were grafted to amputated limb stumps (Figure 4A). As anticipated, neither grafted EB blastemas (25 of 30) or apical LB blastemas (14 of 17) resulted in formation of ectopic structures (Figure 5B,D). As was observed when these cells were grafted to amputated limb stumps (Figures 2 and 4), the grafted GFP-positive cells survived and their progeny integrated into the host site but were not “resorbed” or lost.

To test the hypothesis that a functional nerve is required to maintain blastema cells as positionally labile (undifferentiated), we repeated this experiment by grafting blastemas from limbs in which we severed the brachial nerves (denervated) of the donor limbs three days prior to grafting. In contrast to grafts of either an EB or apical-LB blastema from innervated limbs, these same grafts from denervated limbs resulted in the formation of ectopic limb structures (Figures 5C,E, 9 of 14 EB blastemas and 6 of 9 apical LB blastemas). These structures were sometimes segmented and terminated in a segment that tapered to form what appeared to be a digit tip (arrows in Figure 5C, E). We interpret these results to indicate that when nerve signals are lost (denervation), blastema cells begin to differentiate and acquire stable positional information that corresponds to their position in the limb, which in both the EB blastema and the apical region of later stage blastemas corresponds to the distal tip of the limb [8,56]. Examples of the types of ectopic growth phenotypes observed in this study are illustrated in Figure S4, and quantified in Table S1.

## Discussion

### Positional information is liable in EB and apical-LB blastema cells

**Figure 6:**
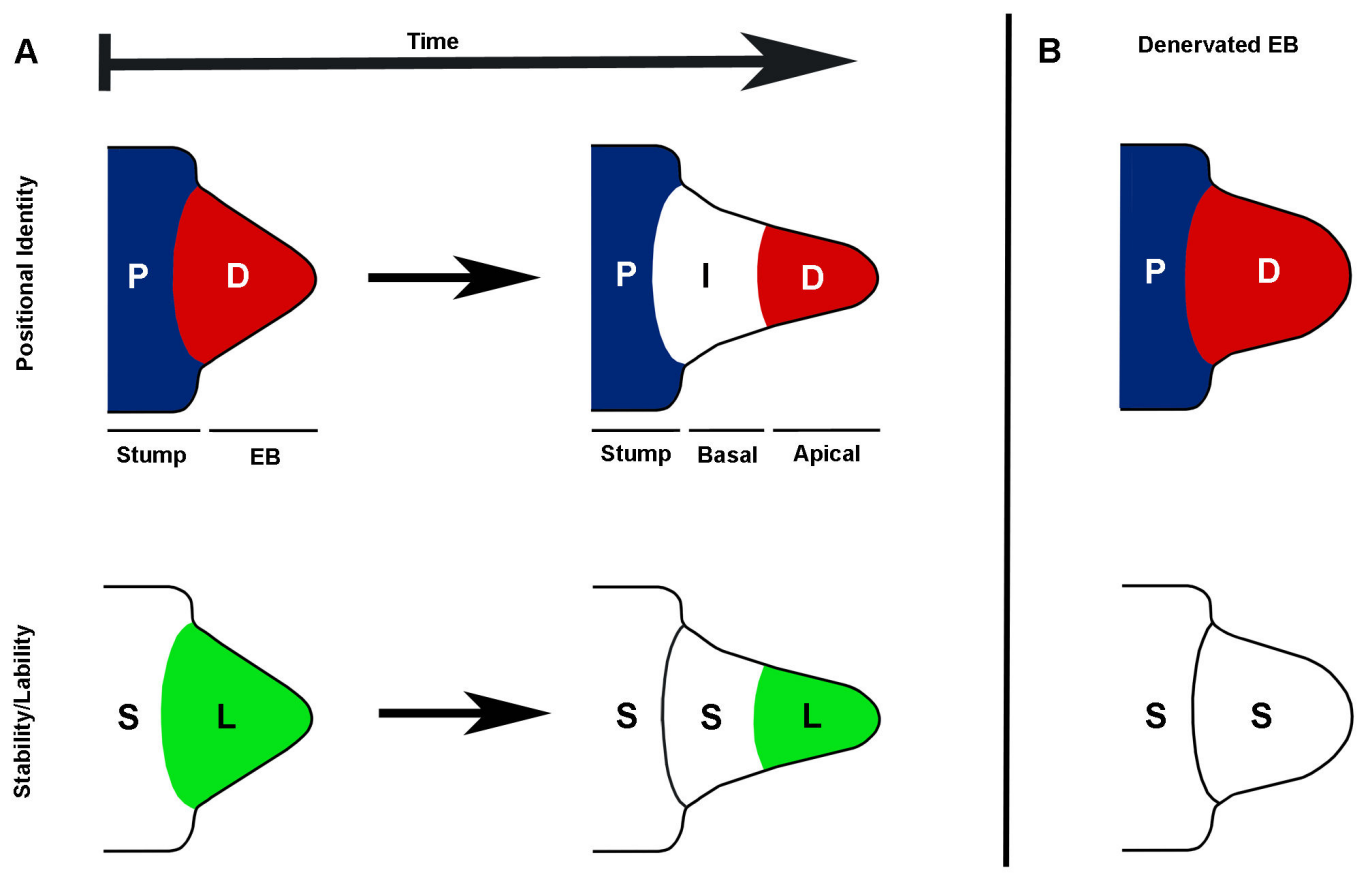
Lability of positional information was nerve dependent in the limb blastema. (**A**) (top panel) The “Distal-First” hypothesis is based on the idea that the early blastema is composed entirely of cells with the distal-most (“D”) identity. The intermediate (corresponding to the pattern between the distal tip and the proximal level of amputation) positional information (“I”) is intercalated as these distal cells interact with the proximal information (“P”) in the stump. (**A**) (bottom panel) The distal positional information in the early blastema and the apical-tip of the late blastema is labile (“L”). The proximal and intermediate information in the stump and basal region of the late blastema, respectively, is stabile (“S”). (**B**) Removal of signals from the nerve by denervation results in the loss of lability and premature stabilization of the distal-most positional information before the intermediate identities have been intercalated.

The process of regeneration requires that cells from the remaining structure, the amputated limb stump (proximal), establish the new pattern of the missing structure (distal) as articulated by Rose’s rule of distal transformation [37]. It follows that for this to occur, cells with proximal positional information need to be reprogramed such that they (as well as their progeny) can acquire distal positional information. Our finding that the early blastema and apical tip of the late blastema are positionally labile is consistent with the hypothesis that these cells are being reprogrammed so as to acquire new positional information. The additional observation that the basal region of the late blastema is positionally stable indicates that as blastema cells begin to redifferentiate, their newly acquired positional identity becomes stabilized (Figure 6).

Results from previous experiments using exogenous retinoic acid (RA) to reprogram the positional information of limb blastema cells are consistent with this hypothesis. Limb cells are differentially responsive to RA treatment depending the stage of regeneration during which they are exposed [57]. Early-mid bud stage blastemas exhibit the strongest reprogramming phenotypes, while late stage blastemas are only reprogrammed distally, and even later “differentiation” stage blastemas as well as pre-blastema stages are unaffected. These RA-responsive stages coincide with the presence of the cells we have identified as being positionally labile (EB and apical LB blastema cells).

Our experiments do not address the issue of whether or not positionally labile blastema cells have positional information. It is likely that they do since early blastema cells express a HOX code corresponding to the distal tip of the limb pattern [8]. This distal HOX code is expressed in EB blastemas regardless of their position along the proximal-distal limb axis, indicating that the HOX code is not stable in the early blastema [8]. *Hoxa-9* and *Hoxa-13* (a distal marker) are expressed simultaneously in the early blastema mesenchyme, after which *Hoxa-13* becomes distally restricted to the apical blastema cells at the medium bud and later stages [8]. Thus cells in the basal region of later stage blastemas acquire a new, more proximal HOX code, which based on the results of the current study, becomes stabilized as the cells begin to redifferentiate.

An alternative interpretation of the data from the present study is that although grafted EB and apical LB blastema cells are not “resorbed” or lost after grafting [17-23], they have distal positional information that is stable, i.e. that their positional information is not labile. This interpretation is based on the “Distal-First” hypothesis [8,9,56,58] discussed above. By this alternative view, the cells of a proximal EB blastema as well as from the apical region of a proximal LB blastema would maintain their distal identity after grafting to a distal host limb, would differentiate as distal structures, and would not induce supernumerary limb pattern. Although the results from grafting of blastemas to amputated limb stumps (as was done in the classic experiments as well as in our present study, Figure 2A,B, and Figure 4A,B) do not allow us to distinguish between these two interpretations (positionally labile or positionally stable), our new, additional experiments (cell contribution from grafted blastemas, grafting of FL blastema cells from HL stumps, and grafting of blastemas to lateral wounds) are most consistent with the conclusion that these cells are positionally plastic/labile.

We observed that the cells from grafted distal EB blastemas as well as from the apical region of distal LB blastemas survived post-grafting and contributed to a variety of tissues at more proximal levels in the regenerate. The phenomenon of distal-to-proximal contribution has been reported previously [5,39], though as noted above the lack of molecular markers at present does not allow us to know if the blastema cells with distal positional information contribute to the formation of more proximal pattern. Secondly, FL blastema cells grafted to a HL host are induced to increase expression of *Tbx4* (HL marker) and to decrease expression of *Tbx5* (FL marker), indicating that EB blastema cells can be reprogrammed in response to signaling associated with the HL host tissues. We note that these data do not address the mechanisms whereby FL/HL and Proximal/Distal positional information are specified, but rather that this information is labile and can be reprogrammed. Finally, neither distal EB blastemas nor the apical region of LB blastemas make ectopic structures when grafted to a lateral wound (Figure 5B, D), even though they have the developmental potential to do so if the donor limb is denervated prior to grafting (Figure 5C, E). Taken together, these data are most consistent with a model in which EB and apical LB blastema cells have distal positional information, but this information is labile and can be reprogrammed in response to position-specific cues in the host microenvironment (Figure 6).

Lastly, it is also possible that a community, or threshold, effect may play a role in the specification of positional information during regeneration as has been observed in embryos [59]. In this instance, we envision that positional information of small grafts could be more easily altered than in large grafts of blastema tissue. While future work will help determine this possibility, we do not think that a community effect was responsible for the differences we observed in the current study. First, all of our grafting studies were performed using EB, apical-LB and basal-LB tissue grafts of similar size. Live images of these grafts 1-week post grafting in Figures 1, 4, and 5 documented that the size was consistent among the different tissues. Second, we observed that denervated EB and apical-LB tissue grafts induced the formation of ectopic limb structures when grafted into a lateral wound, while the innervated EB and apical-LB grafts (obtained from the contralateral forelimb) did not. This observation cannot be explained by a threshold effect.

## Conclusions

Based on our findings, it is evident that in addition to being a mixture of limb progenitor cells from different lineages [39,40], blastema cells also are dynamically heterogeneous in terms of being positionally stable or labile. Recognizing this temporal and spatial heterogeneity is important in terms of designing experiments and collecting samples in order to understand how positional information is regulated during limb regeneration.

Finally, although the mechanism by which positional identity is regulated is unknown, given that epigenetic modifications play an important role in the specification and differentiation of cells in embryos, stem cell cultures, and tumors, we assume that a similar mechanism acts during regeneration to stabilize the positional identity of blastema cells as they redifferentiate [60-62]. It also appears that regardless of the mechanism, the loss, reacquisition and stabilization of positional identity is regulated by signaling from the nerve and AEC (reviewed in [63]). By this view (Figure 6), apical blastema cells in proximity to the AEC would be maintained in a positionally labile (undifferentiated) stage; whereas cells at more proximal regions of the blastema would no longer be influenced by nerve/AEC signaling and would acquire a new positional identity corresponding to the adjacent stump tissues as they begin to redifferentiate.

## Supporting Information

Figure S1 **Cartoons representation of the surgical manipulations and assays performed in this study.** (**A**) Blastema tissue grafts from a proximal donor site to a distal host location. In these manipulations we assayed the ability of the grafted tissues to generate limbs with duplicated proximal/distal structures. This assay was performed in the studies shown in Figures 2A, 2B, 4A, and 4B. (**B**) Blastema tissue grafts from a distal donor site to a proximal host location. In these manipulations we assessed whether the grafted tissues lived and differentiated into tissues in the regenerate. This assay was performed in the studies depicted in Figures 2D-I and 4C-J. (**C**,**D**) Forelimb EB blastemas were grafted to a forelimb host location (**C**) or hind limb host location (**D**). The blastemas were harvested at LB stage, the grafted GFP+ cells were sorted from the host GFP- cells by FACS, and molecular analysis was performed on the sorted populations. These manipulations were used in the experiment described in Figure 3. (**E**) Blastema or mature tissues were grafted into a lateral wound with a nerve deviation, and assayed for the ability to induce the formation of ectopic cartilage structures [11]. This assay was used in the study depicted in Figure 5.
(TIF)

Figure S2 **Scatter plots and histograms of FAC sorted blastema cells.** Scatter plots and fluorescent histogram of FAC-sorted blastema cells from a GFP+ transgenic animal (**A**), a white GFP- animal (**B**), and a mosaic blastema with GFP+ and GFP- cells (**C**). The initial gate was based on forward and side scatter to separate blastema cells from cellular debris (left plots). These cells were further gated based on the intensity of green fluorescence (right plots). The histograms represent the distribution of cells according to their mean fluorescent intensity (bottom panel). The mean fluorescent intensity of GFP- and GFP+ cells is between 1 and 10, and 10^3^ and 10^4^ relative units, respectively. The plots shown in (**C**) are from a FL grafted to HL experimental replicate from the study described in Figure 3.
(TIF)

Figure S3 **GFP+/GFP- doublet discrimination in mosaic blastema populations.** Scatter plots are the combined data from three independent samples (8 blastemas/sample) of GFP+ and GFP- blastema cells. The blastemas were dissociated exactly as described in materials and methods. (**A**) The plot depicts the pulse width (FL1-W) *versus* area (FL1-A) of each particle detected in the GFP channel (FL1, 488 nm laser excitation, and 530 nm fluorescence detection with 30 nm band-width). The average pulse width of a single particle was 326 relative units. Doublets, which should be roughly twice the size of a single cell, had an average pulse width of 781 relative units. To ensure that we included all of the doublets in our calculation, we counted the particles from 500 to >1000 relative FL-W units. 1.36% +/- 0.311% (SEM) of the GFP+ population appears to be GFP+/GFP- doublets. (**B**) The plot depicts the height (i.e. intensity of fluorescence pulse) (FL1-H) *versus* the area (FL1-A) of the pulse detected in GFP channel (FL1). Since the average intensity of fluorescence from a GFP+/GFP- doublet will be less than a single GFP+ or GFP+/GFP+ doublet, doublets that contain a contaminating GFP- host cell will fall to the left of the prominent population. By this method, we determined that 0.0371% +/- 0.023% (SEM) of the GFP+ population are GFP+/GFP- doublets. We conclude that a minimal amount of GFP+/GFP- doublets was included in our molecular analysis described in Figure 3.
(TIF)

Figure S4 **Ectopic cartilage phenotypes observed when tissue is grafted into an Accessory Limb Assay.** Whole mount bone (red) and cartilage (blue) preparations were performed 9 weeks post-surgery. (**A**) Some of the grafts into a lateral wound that did not result in the formation of ectopic cartilage or bone. The red arrow indicates the location of the surgical manipulation. (**B**) Some grafts resulted in the formation of single cartilage elements (red arrow). (**C**) Some grafts resulted in the formation of structures that had multiple (2 or more) elements and were symmetrical. The depicted example of this subgroup has 4 skeletal elements, indicated by 4 arrows. (**D**) Some grafts resulted in the formation of structures that were similar to a complete limb, which had multiple skeletal elements with asymmetry. Further quantification of these subgroups from the study described in Figure 5 is presented in Table S1.
(TIF)

Table S1 **A detailed breakdown of the morphology of ectopic cartilage growths from tissue grafts into a lateral wound.**
(XLSX)
