# Supplementary figures and images for: Positional Information Is Reprogrammed in Blastema Cells of the Regenerating Limb of the Axolotl (Ambystoma mexicanum)
Source: PLoS One. 2013 Sep 27;8(9):e77064. doi: 10.1371/journal.pone.0077064 (PMC3785456; doi:10.1371/journal.pone.0077064)

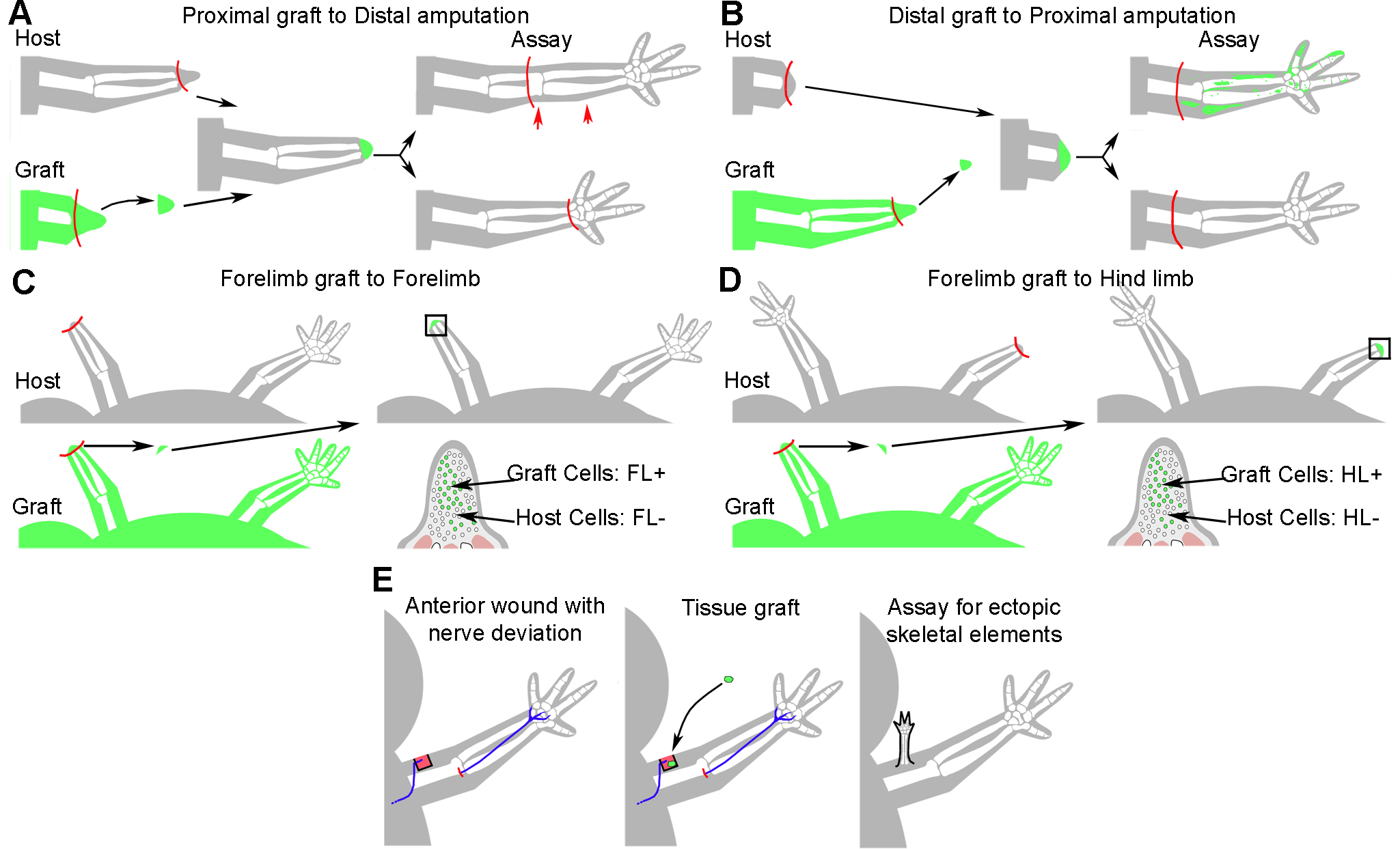

Supplement: Figure S1 — Cartoons representation of the surgical manipulations and assays performed in this study. (A) Blastema tissue grafts from a proximal donor site to a distal host location. In these manipulations we assayed the ability of the grafted tissues to generate limbs with duplicated proximal/distal structures. This assay was performed in the studies shown in Figures 2A, 2B, 4A, and 4B. (B) Blastema tissue grafts from a distal donor site to a proximal host location. In these manipulations we assessed whether the grafted tissues lived and differentiated into tissues in the regenerate. This assay was performed in the studies depicted in Figures 2D-I and 4C-J. (C,D) Forelimb EB blastemas were grafted to a forelimb host location (C) or hind limb host location (D). The blastemas were harvested at LB stage, the grafted GFP+ cells were sorted from the host GFP- cells by FACS, and molecular analysis was performed on the sorted populations. These manipulations were used in the experiment described in Figure 3. (E) Blastema or mature tissues were grafted into a lateral wound with a nerve deviation, and assayed for the ability to induce the formation of ectopic cartilage structures [11]. This assay was used in the study depicted in Figure 5. (TIF) [file pone.0077064.s001.tif]

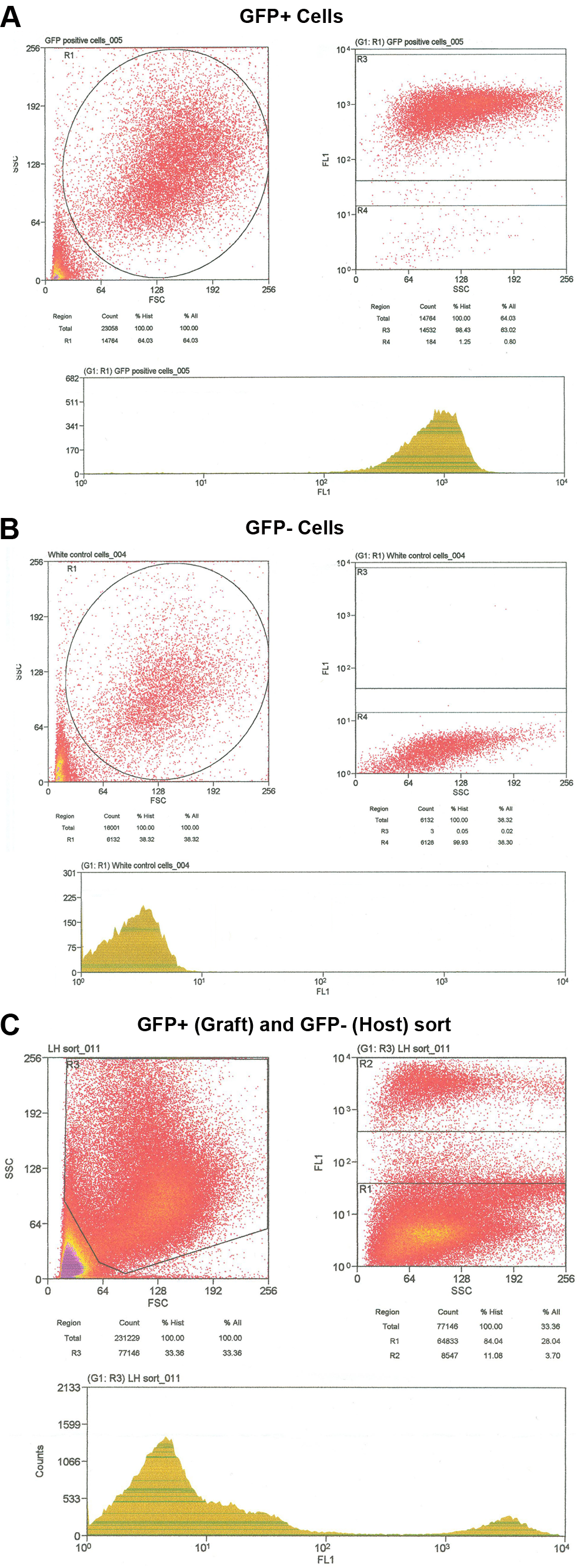

Supplement: Figure S2 — Scatter plots and histograms of FAC sorted blastema cells. Scatter plots and fluorescent histogram of FAC-sorted blastema cells from a GFP+ transgenic animal (A), a white GFP- animal (B), and a mosaic blastema with GFP+ and GFP- cells (C). The initial gate was based on forward and side scatter to separate blastema cells from cellular debris (left plots). These cells were further gated based on the intensity of green fluorescence (right plots). The histograms represent the distribution of cells according to their mean fluorescent intensity (bottom panel). The mean fluorescent intensity of GFP- and GFP+ cells is between 1 and 10, and 103 and 104 relative units, respectively. The plots shown in (C) are from a FL grafted to HL experimental replicate from the study described in Figure 3. (TIF) [file pone.0077064.s002.tif]

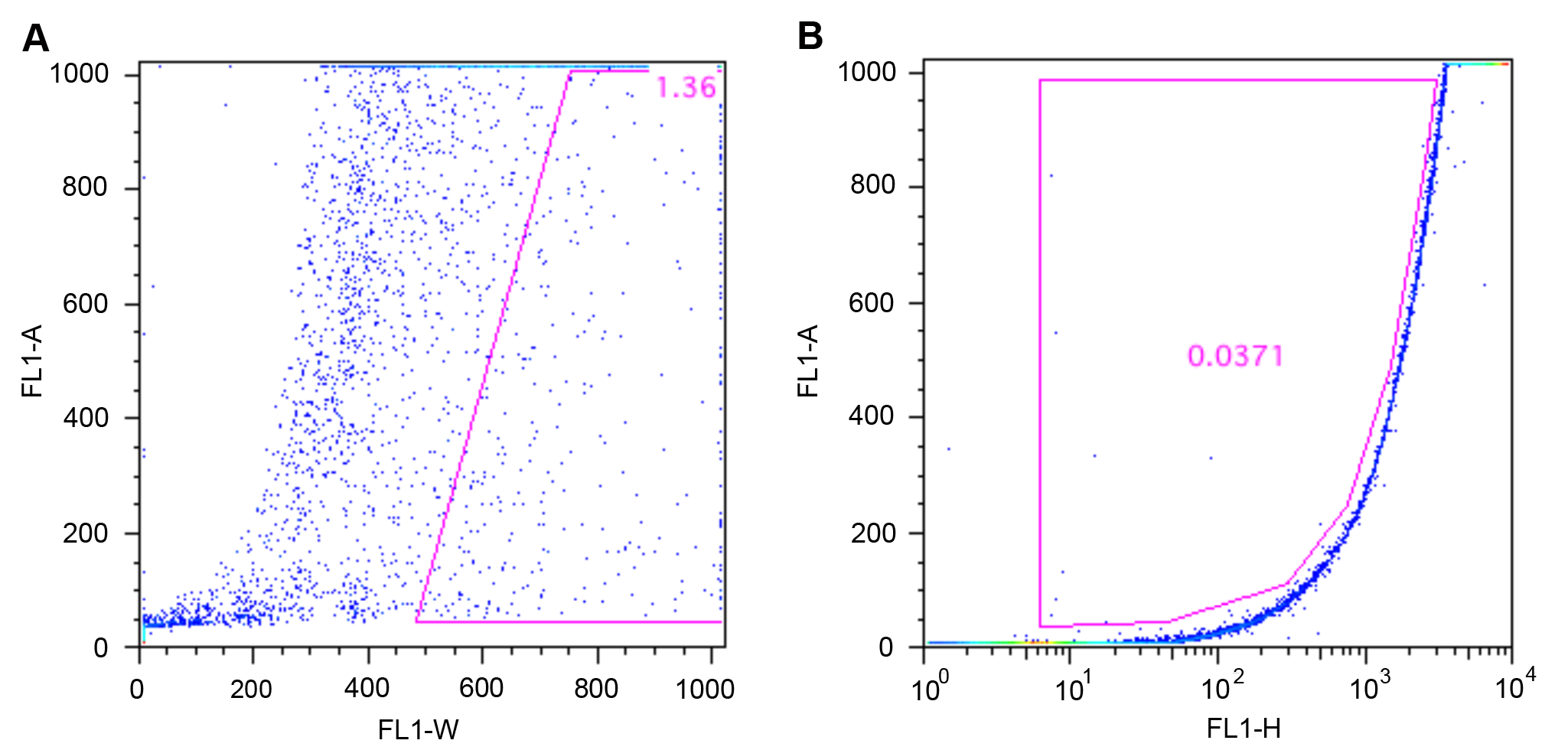

Supplement: Figure S3 — GFP+/GFP- doublet discrimination in mosaic blastema populations. Scatter plots are the combined data from three independent samples (8 blastemas/sample) of GFP+ and GFP- blastema cells. The blastemas were dissociated exactly as described in materials and methods. (A) The plot depicts the pulse width (FL1-W) versus area (FL1-A) of each particle detected in the GFP channel (FL1, 488 nm laser excitation, and 530 nm fluorescence detection with 30 nm band-width). The average pulse width of a single particle was 326 relative units. Doublets, which should be roughly twice the size of a single cell, had an average pulse width of 781 relative units. To ensure that we included all of the doublets in our calculation, we counted the particles from 500 to >1000 relative FL-W units. 1.36% +/- 0.311% (SEM) of the GFP+ population appears to be GFP+/GFP- doublets. (B) The plot depicts the height (i.e. intensity of fluorescence pulse) (FL1-H) versus the area (FL1-A) of the pulse detected in GFP channel (FL1). Since the average intensity of fluorescence from a GFP+/GFP- doublet will be less than a single GFP+ or GFP+/GFP+ doublet, doublets that contain a contaminating GFP- host cell will fall to the left of the prominent population. By this method, we determined that 0.0371% +/- 0.023% (SEM) of the GFP+ population are GFP+/GFP- doublets. We conclude that a minimal amount of GFP+/GFP- doublets was included in our molecular analysis described in Figure 3. (TIF) [file pone.0077064.s003.tif]

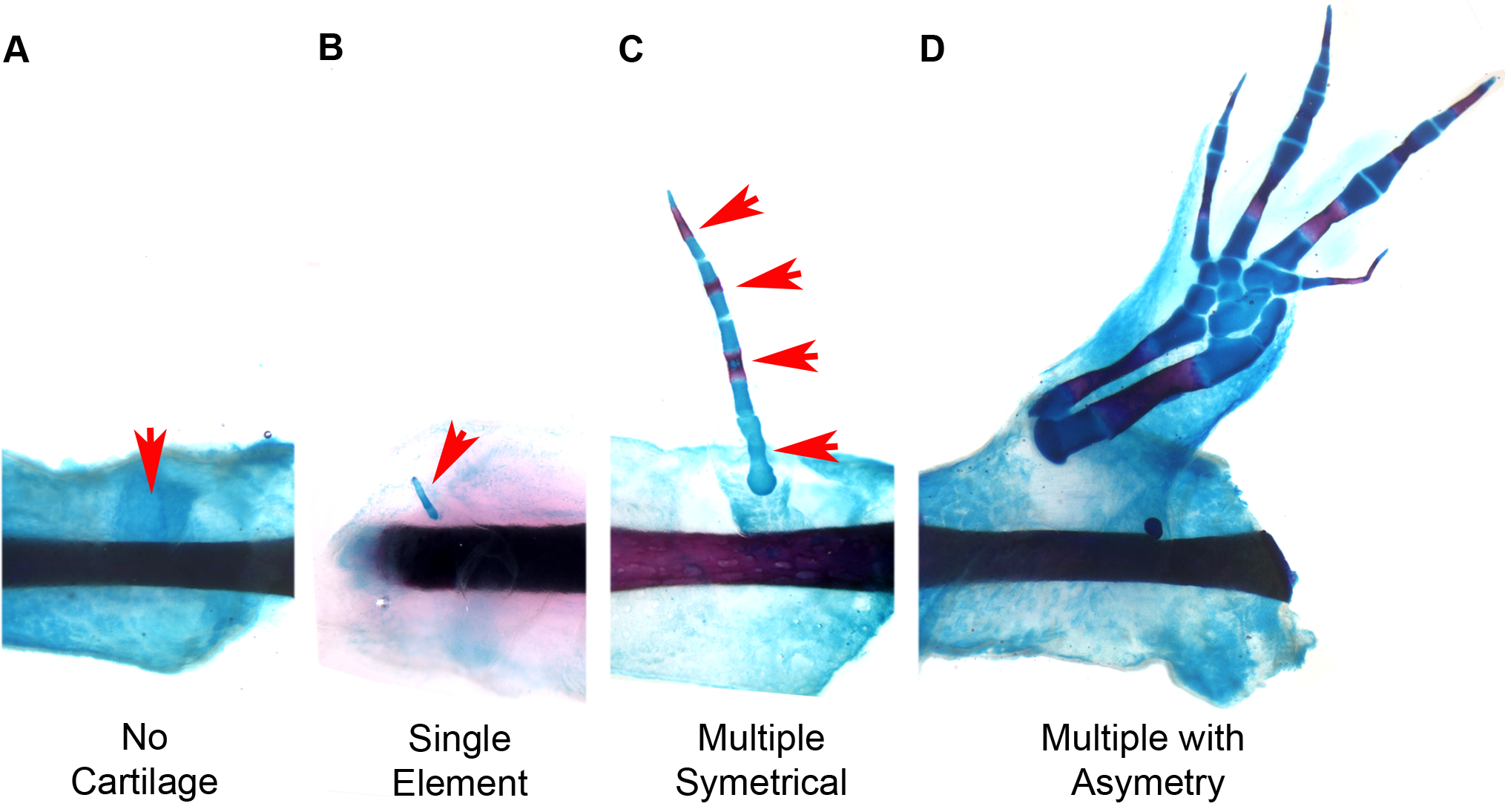

Supplement: Figure S4 — Ectopic cartilage phenotypes observed when tissue is grafted into an Accessory Limb Assay. Whole mount bone (red) and cartilage (blue) preparations were performed 9 weeks post-surgery. (A) Some of the grafts into a lateral wound that did not result in the formation of ectopic cartilage or bone. The red arrow indicates the location of the surgical manipulation. (B) Some grafts resulted in the formation of single cartilage elements (red arrow). (C) Some grafts resulted in the formation of structures that had multiple (2 or more) elements and were symmetrical. The depicted example of this subgroup has 4 skeletal elements, indicated by 4 arrows. (D) Some grafts resulted in the formation of structures that were similar to a complete limb, which had multiple skeletal elements with asymmetry. Further quantification of these subgroups from the study described in Figure 5 is presented in Table S1. (TIF) [file pone.0077064.s004.tif]
